# Screening of Genes Related to Growth, Development and Meat Quality of Sahan Crossbred F1 Sheep Based on RNA-Seq Technology

**DOI:** 10.3389/fvets.2022.831519

**Published:** 2022-04-07

**Authors:** Yali Song, Quanwei Zhang, Jinping Shi, Lingjuan Fu, Shuru Cheng

**Affiliations:** ^1^College of Animal Science and Technology, Gansu Agricultural University, Lanzhou, China; ^2^College of Life Science and Biotechnology, Gansu Agricultural University, Lanzhou, China

**Keywords:** differentially expressed genes, growth and development, meat quality, immunofluorescence, sheep, transcriptome sequencing

## Abstract

This study aimed to identify genes related to sheep growth, development and meat quality. Small-tailed Han sheep (STH), and small-tailed Han sheep and Suffolk crossbred F1 (STH×SFK), were selected to determine the growth performance, slaughter performance, and meat quality. The *longissimus dorsi* muscle was selected for transcriptome sequencing, and the target gene was screened based on bioinformatics analysis; real-time fluorescent quantitative PCR (RT-PCR) and western blotting (WB) were conducted to verify the target gene. Locations of genes in tissues were confirmed via immunofluorescence. The results showed that the pre-slaughter live weight, bust circumference, slaughter performance, and marbling score of the STH×SFK population were significantly higher than those of the STH population (*P* < 0.01). Sequencing results showed that 560 differentially expressed genes (DEGs) were identified in the STH×SFK population, of which 377 exhibited up-regulated and 183 exhibited down-regulated expression levels. GO annotation revealed that DEGs could be classified into 13 cell components, 10 molecular functions, and 22 biological processes. The KEGG enrichment analysis showed that DEGs were mainly enriched in the Rap1 signaling pathway, Ras signaling pathway, and other pathways related to growth and meat quality. Based on the GO and KEGG analyses, four candidate genes related to sheep growth and meat quality, namely myostain (*MSTN*), interferon-related developmental regulator 1 (*IFRD1*), peroxisome proliferator activator receptor delta (*PPARD*), and myosin light chain 2 (*MLC2* or *MYL2*), were screened. The expression levels of genes and proteins were verified via RT-PCR and WB, and the results were consistent with the trend of transcriptome sequencing. Immunofluorescence results showed that IFRD1 was expressed in the cytoplasm and nucleus, and MYL2 was expressed in the cytoplasm. This study revealed the mechanism of gene regulation of sheep growth and development at the molecular level and provided a theoretical basis for studying sheep genetics and breeding.

## Introduction

Sheep is considered one of the earliest domesticated domestic animals; additionally, after subjection to a long period of natural selection and artificial breeding, the formation of a rich sheep germplasm resource occurs. Lamb consumption is popular because of its high protein and low-fat content. Although China presents with a substantial population involved in mutton consumption and sheep breeding (1), China's domestic sheep breeds demonstrate lower meat yields and poorer meat quality compared with the exotic breeds. Excellent exotic breeds are often introduced for cross-breeding purposes to rapidly improve the production performance and meat quality of local sheep breeds and can help overcome issues encountered with pure breeds. The quality of sheep meat is restricted (2–4). Small-tailed Han sheep are known for their high fertility and are characterized by early maturity and rough feeding resistance. Suffolk sheep constitute one of the world's largest populations of sheep breed used for obtaining meat, exhibiting a sturdy physique and weight. Owing to its fast growth and development, high lean meat rate, and heterosis, it is used as the terminal crossbred male parent for the production of carcasses and high-quality lamb meat (5). Studies have demonstrated that when small-tailed Han sheep are subjected to crossing with Dorper and Mongolian sheep, the bust, live weight, and net meat weight of Dorper crossbred F1 and Mongolian sheep crossbred F1 are found to be significantly higher than those of small-tailed Han sheep (6, 7). Therefore, the consideration of heterogeneity can increase the coverage of improved varieties, and exert an impact on commodity and economic value, as well as promote the development of animal husbandry.

Transcriptome sequencing (RNA-seq) is a large-scale sequencing technology that can be used to comprehensively and rapidly obtain almost all transcripts of a specific organ or tissue in a certain state. It can not only help detect transcripts corresponding to the existing genome sequences but can also help discover and quantify new transcripts, thus emerging as an important tool for gene expression and transcription analysis (8). It has been widely used in the study of the molecular mechanisms of important economic traits in livestock and poultry (9, 10). Yao et al. (11) have reported the use of the whole transcriptome to sequence high- and low-breed Bame mutton sheep to study litter size-related genes, and have identified genes related to reproduction, such as *JUN, ITPR3*, and *HERC5*. Liu et al. (12) used RNA-Seq technology to study the miRNAs of prenatal and postpartum skeletal muscles of sheep, and performed screening of a total of 1126 miRNAs, of which 40 were new candidate miRNAs, following which they confirmed that the development of skeletal muscle was related to the targeted genes of miRNA. Shi et al. (13) conducted a transcriptome analysis and comparison of the *longissimus dorsi* muscle of different sheep and performed screening of 1,445 DEGs, of which 199 were related to growth and development and 43 were key genes related to meat quality traits. Zhan et al. (14) performed whole transcriptome sequencing using the *longissimus dorsi* muscle tissue of sheep in the prenatal and neonatal stages and identified 3276 DEGs, which were found to be significantly enriched in 22 pathways. Among them, the Rap1 signaling pathway was deemed the most significant, and down-regulated genes were significantly enriched in 17 pathways, mainly in the AMPK pathway. Several studies have reported the analysis of the heat stress of sheep by transcriptome and have noted that it can induce changes in the expression of the hypothalamus-related genes; additionally, it has been reported that they can affect the growth and development of sheep through the calcium signaling pathway (15). Other studies have reported the use of transcriptomics to analyze the skeletal muscles of DP and STH sheep populations, and discovered genes related to muscle growth and development, such as *ITGBL1, HPSE*, and *APOBEC3G* (16). Although there are several studies available on sheep transcriptome analysis, most studies have focused on the screening of genes whose expression levels affect a certain trait, and there are few reports available on crossbred sheep, which cannot provide genetic data for improving the overall performance of sheep. Therefore, elucidation of the regulatory mechanisms that affect the performance of crossbred sheep at the molecular level presents with important breeding significance.

In this study, the transcriptome analysis of the *longissimus dorsi* of small-tailed Han sheep (STH), and that of small-tailed Han sheep and Suffolk crossbred F1 (STH×SFK), was performed. The DEGs related to growth, development, and meat quality were screened by combining production performance and meat quality, to identify new genes for sheep molecular breeding, to optimize sheep growth and meat quality traits, and to accelerate the rate of sheep breeding.

## Materials and Methods

### Experimental Animals, Index Determination, and Sample Collection

In this study, 7 ± 2-day-old healthy STH (*n* = 50) and STH×SFK (*n* = 50) lambs with no significant difference in body weight were selected as experimental animals and were obtained from the Pingshan Lake breeding farm (Ganzhou District, Zhangye City, Gansu Province, China). To ensure the same feeding management and nutritional level, lambs were weaned at 2 months of age. All lambs were fed with concentrate three times a day (7:00, 12:00, and 19:00), and hay and water were provided *ad libitum*. At the age of 6 months, body size (body height, body length, and bust circumference) was measured before slaughter. Six STH and STH×SFK sheep were randomly selected for slaughter, and the slaughter performance (pre-slaughter live weight, slaughter rate, carcass weight, net meat weight, meat-to-bone ratio, eye muscle area, back meat thickness) and meat quality (water loss rate, cooking loss, tenderness, pH value, marbling) were measured (17, 18). Immediately after slaughter, STH and STH×SFK *longissimus dorsi* were removed, and a segment was placed in a cryotube that had been subjected to high-pressure treatment in advance, following which the sample was marked and rapidly frozen in a liquid nitrogen tank. The samples were stored in a refrigerator at −80°C for subsequent transcriptome sequencing and RT-PCR analysis. Another segment was selected and subjected to fixation in a 4% paraformaldehyde solution for obtaining paraffin-embedded sections. The data on production performance, slaughter performance, and meat quality data of the two cross-bred sheep populations were sorted using Excel tables. SPSS (version 22.0; SPSS Inc., Chicago, IL, USA) was used to perform an independent-sample t-test using the data, and the results have been expressed as X ± SD. Statistical significance was set at *p* < 0.05.

### Hematoxylin and Eosin Staining (H&E)

For analysis via staining, the nuclei of the tissue cells were subjected to staining with hematoxylin to obtain a bright blue color; the cytoplasm was subjected to staining with eosin to achieve a pink to peach red color gradient with different shades.

Furthermore, the paraffin-embedded sections were placed in a 60°C oven for 1–2 h, following which they were dewaxed using water and conventional xylene and ethanol; then, the nucleus was subjected to staining with hematoxylin for 5–10 min, following which rinsing steps were performed with running water and differentiation was observed using hydrochloric acid and alcohol treatment performed for a few seconds; thereafter, the sections were rinsed in running water for ~10 min, followed by subjection to staining with eosin for 30 s to 60 s. Then, they were rinsed with running water and dehydrated with gradient alcohol. After wiping with absorbent paper, the sheet was sealed with neutral resin, following which it was dried and observed under a microscope.

### cDNA Library Construction and Transcriptome Sequencing

Total RNA was extracted from STH and STH×SFK (*n* = 6 for each group) sheep *longissimus dorsi* muscle using Trizol reagent. After ensuring quality control, the *longissimus dorsi* muscle mRNA was enriched with magnetic beads with Oligo (dT) and a strand of cDNA was randomly synthesized using the mRNA as a template; complementary two-stranded cDNA was synthesized, double-stranded cDNA was purified, end repair was performed, sequence adapters were used, and AMPure XP beads (Beckman Coulter, Beverly, MA, USA) were used for fragment size selection (length, 200–500 bp). Finally, PCR amplification was performed to construct the cDNA library. After the library was validated, the Illumina high-throughput sequencing platform (HiSeq/MiSeq) was used for sequencing. Transcriptome sequencing was performed by the Guangzhou Saizhe Biological Company (Guangzhou, China).

### Screening of DEGs and Functional Annotation

Clean reads obtained via sequencing were filtered, remove linkers, remove reads that contain more than 10% N and low-quality reads(the number of bases with quality value SQ ≤ 20 accounts for more than 50% of the entire read) to obtain high-quality clean reads and then were aligned to the sheep reference genome (*Oar._v. 4.0*) using Bowtie 2 and TopHat 2 (version 2.0.3.12) (19, 20) sequence software to obtain the alignment results of each sample; Cufflinks was used for gene assembly, and Cuffmerge (21) was used to merge the assembly results of multiple samples to obtain information on a complete gene.

For determining the expression level of the transcript, the FPKM algorithm is used for standardization processing (22) to compare the differences in gene expression levels between different varieties. FPKM ≥ 0.1 was used as the threshold for judging gene expression (23). Using FDR < 0.01 and |log2FC|>1 as the standard (15, 23) DEGs were screened, following which DEGs were annotated and enriched through the Gene Ontology (GO) and Kyoto Encyclopedia of Genes and Genomes (KEGG). We selected the DEGs from the GO terms and pathways including the keywords such as development, growth, muscle, which were involved in sheep production performance and meat quality according to previous studies (6, 7, 13).

### Verification of RT-qPCR Analysis

Total RNA extraction from the *longissimus dorsi* muscle of two sheep populations was performed using a suitable kit, as per the manufacturer's instructions (Vazyme Biotech Co., Ltd., Nanjing); thereafter, RNA samples were used as a template for reverse transcription into cDNA according to the instructions outlined by the manufacturer of the reverse transcription kit (TransGen Biotech Co., Ltd., Beijing). According to the gene sequence provided by NCBI, Primer Premier 5 software was used to design the primers by themselves and the primers were synthesized by Xi'an Qingke Biology Company (Supplementary Table 1). Using cDNA as a template, RT-qPCR analysis was performed considering the transcription levels of *IFRD1, PPARD, MYL2*, and *MSTN* genes. According to the instructions outlined by the manufacturers of the fluorescence quantitative PCR kit (Quanshijin Biotech Co., Ltd., Beijing), the reaction volume used was 20 μL and the following constituted the reaction mixture: 10 mmol/L, 0.5 μL of upstream and downstream primers, 10 μL of qPCR Super Mix, 1 μL of cDNA, and 8 μL of ddH2O. RT-PCR reaction program considered in the present study was as follows: pre-denaturation at 94°C for 30 s, denaturation at 94°C for 5 s, and extension at 60°C for 30 s, 40 cycles. Using the β-actin (β*-actin*) and glyceraldehyde-3-phosphate dehydrogenase (*GAPDH*) genes as internal reference genes, the fluorescence quantitative results were calculated using the 2^−Δ*ΔCt*^ method, and each sample was tested three times.

### Western Blotting (WB)

The *longissimus dorsi* muscles derived from the two populations were subjected to lysis using the RIPA lysis buffer (Solarbio, Beijing, China) containing PMSF, and the supernatant was centrifuged for total protein extraction; thereafter, the total protein concentration was determined. The protein extract was added with 5 × loading buffer, following which it was subjected to denaturation at 95°C for 10 min and storage at −20°C. Sodium dodecyl sulfate polyacrylamide gel electrophoresis (SDS-PAGE) was performed by adding the denatured protein sample, and the wet transfer method was conducted to transfer the target protein onto a PVDF membrane (Millipore CAT, Billerica, MA). Five percent skimmed milk powder was used for blocking with an incubation for 2 h; the mouse IFRD1 primary antibody (Santa Cruz, USA, dilution ratio 1:800), MYL2 primary antibody (Santa Cruz, USA, dilution ratio 1:500), and β-actin primary antibody (Bioss, Beijing, China, dilution ratio 1:4000) were added and incubation was performed overnight at 4°C, following which the membranes were washed with PBST; goat anti-mouse IgG/HRP secondary antibody (Bioss, Beijing, China, 1:5000) was added, and incubation was performed at 37°C for 2 h. Thereafter, PBST was used in washing steps and ECL was used to for color development for further analysis. β*-actin* expression was considered as a control. The Image-Pro Plus 6.0 (Media Cybernetics Co., Rockville, MD, USA) software was used to perform scanning for the protein gray values.

### Immunofluorescence Analysis

After the tissue was routinely deparaffinized in water, heat-induced antigen retrieval method was performed and washing steps with PBS were conducted after the specimen was cooled (3 times, 8 min/time). A blocking solution was used to seal the tissue for 30 min, and then the primary antibody (Santa Cruz, USA, dilution ratio 1:50) was added, following which overnight incubation was performed at 4°C; washing steps were conducted with PBS, followed by addition of an FITC-labeled secondary antibody (Bioss, Beijing, China, 1:300) and incubation for 30 min. Washing steps were performed with PBS 3 times (8 min/time), DAPI was added for staining of the nucleus for 5 min, and washing steps were performed again with PBS. Finally, the slide was mounted with an anti-fluorescence quenching mounting plate and images were acquired under a fluorescence microscope for observation.

## Results

### Comparison of Growth Performance, Slaughter Performance, and Meat Quality Between STH and STH×SFK

**Figure 1:**
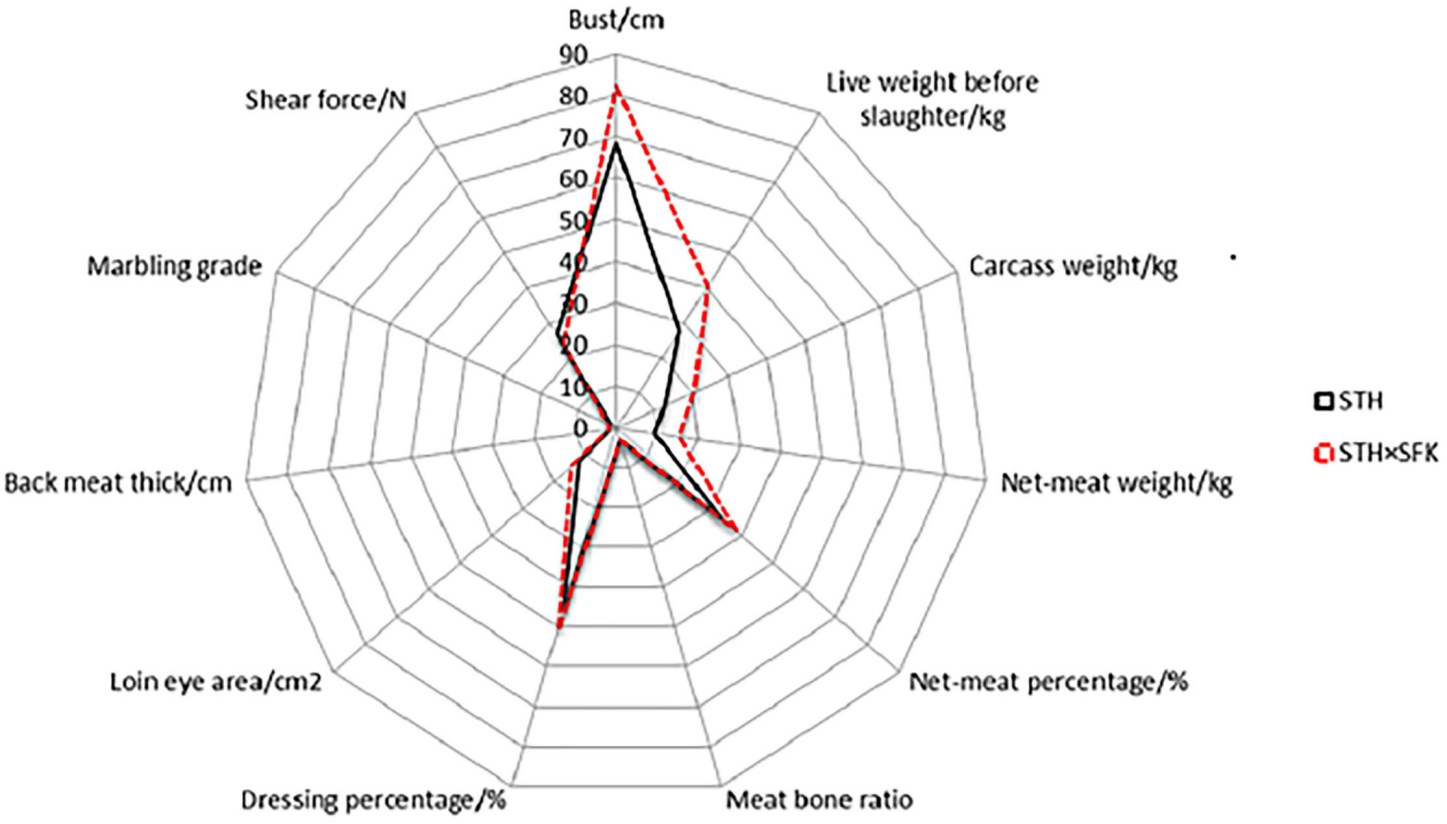
The difference in production performance and meat quality of STH and STH×SFK sheep.

The growth performance, slaughter performance, and meat quality of STH and STH×SFK were presented in Supplementary Table 1; Figure 1. The results showed that in terms of growth performance, the bust circumference of the STH×SFK crossbred population was significantly higher than that of the STH population (*P* < 0.01), with an average increase of 19.8%; however, there was no significant difference in body height and body length. The pre-slaughter live weight, carcass weight, net meat weight, meat-to-bone ratio, slaughter rate, eye muscle area, and back meat thickness of the STH×SFK crossbred population were significantly higher than those of the STH population (*P* < 0.01), while carcass yield rate was not significantly different.

In terms of meat quality, the marbling score of the STH×SFK crossbred population was significantly higher than that of the STH population (*P* < 0.01), and the shear force was significantly higher than that of the STH population (*P* < 0.05). There were no significant differences between other indicators. In summary, the growth performance, and meat quality of the STH×SFK sheep population were better than those of the STH sheep population.

### Histological Observation

**Figure 2:**
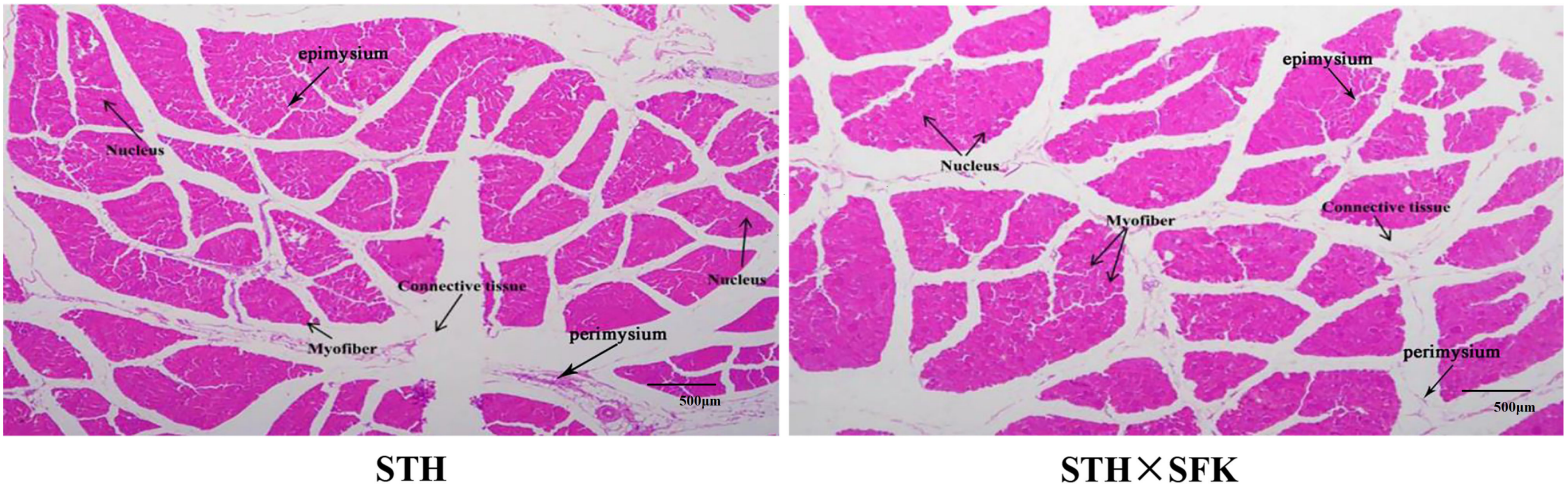
Observation of histological morphology of *longissimus dorsi* muscle (HE ×40).

H&E staining was performed for paraffin-embedded sections of the *longissimus dorsi* muscle of sheep (Figure 2) to observe the histological morphology. The sections of the *longissimus dorsi* muscle tissue of the STH and STH×SFK sheep populations were complete, the muscle cells were separated by white connective tissue, the boundaries between muscle fibers were remarkable, the cell morphology was good, and the nucleus in the middle exhibited marked blue coloration. Compared with the STH sheep population, the STH×SFK sheep population is underdeveloped with connective tissues such as perineum and endomysium.

### Transcriptome Data Analysis

**Figure 3:**
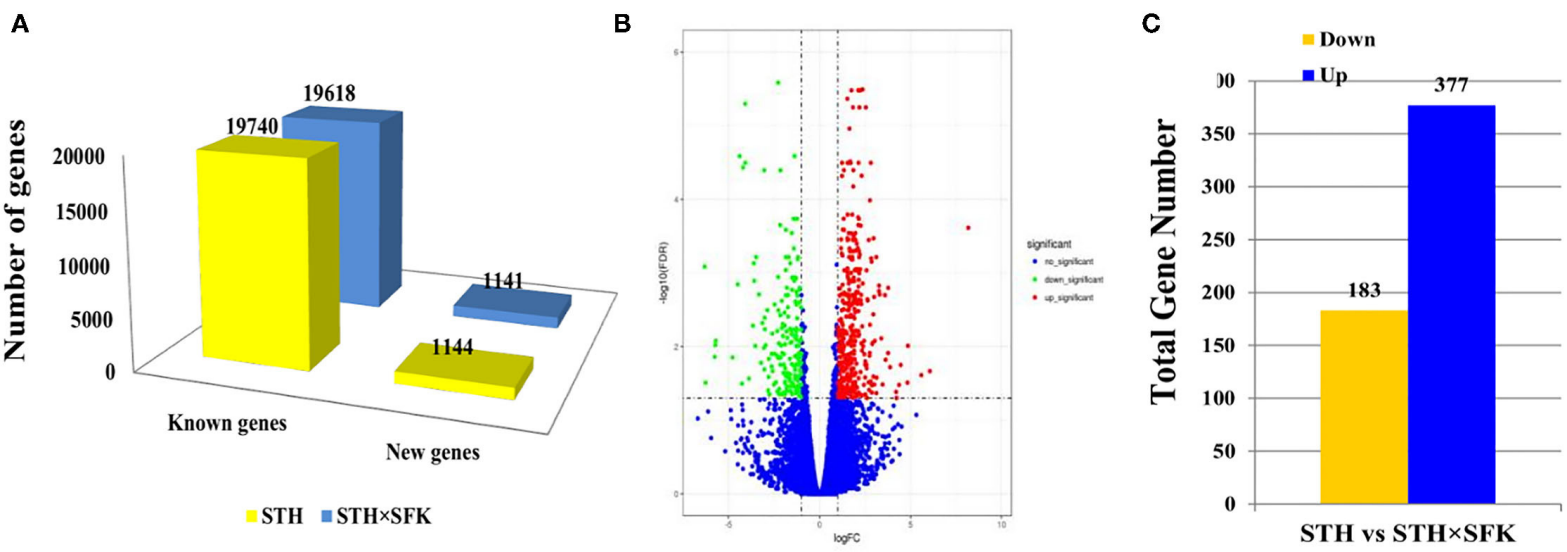
Transcriptome data analysis of *longissimus dorsi* muscle tissue of STH and STH×SFK sheep populations. **(A)** The total number of genes sequenced by the transcriptome in the two sheep groups. **(B)** DEG's volcano map. **(C)** Identify the DEGs of two sheep.

Transcriptome sequencing revealed that STH and STH×SFK presented with an average of 10, 278, 304, 671 and 9, 814, 835, 229 clean data, respectively. Excluding linkers and low-quality sequences, STH and STH×SFK demonstrated an average of 10, 222, 184, 190 and 9, 747, 763, 445 high-quality sequences, respectively, and the Q30 values of the obtained sequences were all >90%. The high-quality sequence was compared to the sheep reference genome (*Oar_v 4.0*), and the comparison rate ranged from 74.42–80.28 %, indicating that the transcriptome sequencing data were accurate and reliable (Supplementary Table 1). In the STH population, 20,884 genes were annotated, including 19, 740 known genes and 1, 144 new genes; in the STH×SFK sheep population, a total of 20, 759 genes were annotated, including 19, 618 known genes and 1, 141 new genes (Figure 3A). Using STH as a control, 560 DEGs were identified (Supplementary Table 2), of which 377 exhibited up-regulated and 183 exhibited down-regulated (Figure 3C) expression levels. To evaluate the overall distribution of DEGs in the two sheep populations, differential multiples and FDR values were used to generate a differentially expressed gene volcano map (Figure 3B).

### GO Annotation and KEGG Analysis of DEGs

**Figure 4:**
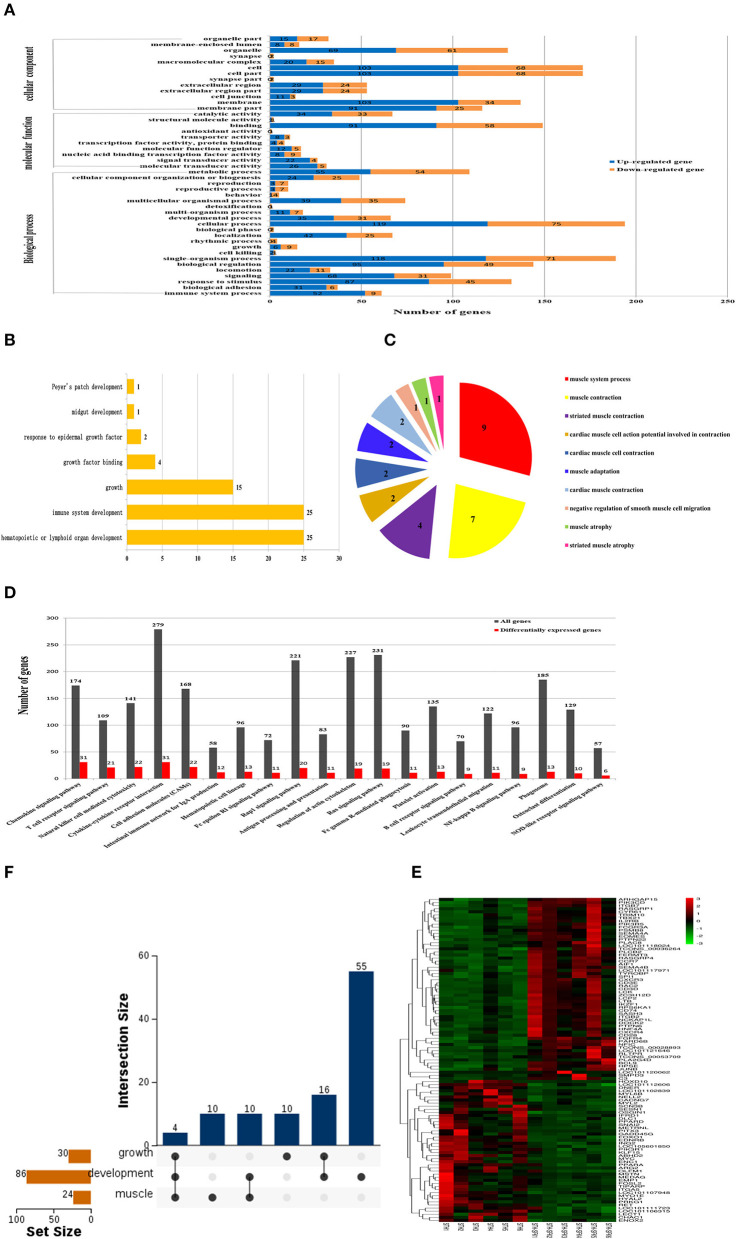
Screening of candidate genes related to growth and meat quality of STH and STH×SFK sheep populations. **(A)** GO functional classification of DEGs (*P* < 0.05). **(B)** GO analysis of candidate DEGs related to sheep growth and development. **(C)** GO analysis of candidate DEGs related to sheep meat quality. **(D)** Determine the important route based on the DEG of two sheep (*P* < 0.05). **(E)** Candidate gene heat map. **(F)** UpSet Venn diagram related to growth and development and meat quality.

Significance analysis showed that the DEGs were significantly enriched in 45 GO entries (*P* < 0.05), including 13 cell components, 10 molecular functions, and 22 biological processes (Figure 4A). Through GO annotation, it was found that the DEGs related to growth and development were significantly enriched in 7 GO items including growth and growth factor binding (Figure 4B), and DEGs related to meat quality were significantly enriched in 10 GO items such as muscular system processes and muscle contractions (Figure 4C). KEGG results revealed that a total of 20 pathways were significantly enriched, especially the Rap1 signaling pathway (20 DEGs), Ras signaling pathway (19 DEGs), and Regulation of actin cytoskeleton pathways (19 DEGs) (Figure 4D). According to the GO annotation and KEGG enrichment results, 86 DEGs were found to be closely related to development, 30 DEGs were related to growth, and 24 DEGs were related to muscle. The results of the heat map construction showed that these DEGs were significantly different in the STH and STH×SFK sheep populations (Figure 4E). Based on the UpSet Venn diagram, four genes co-expressed with *IFRD1, PPARD, MSTN*, and *MYL2* were also discovered for their roles in growth, development, and meat quality (Figure 4F).

### Real-Time Fluorescent Quantitative PCR and Protein Verification of DEGs

**Figure 5:**
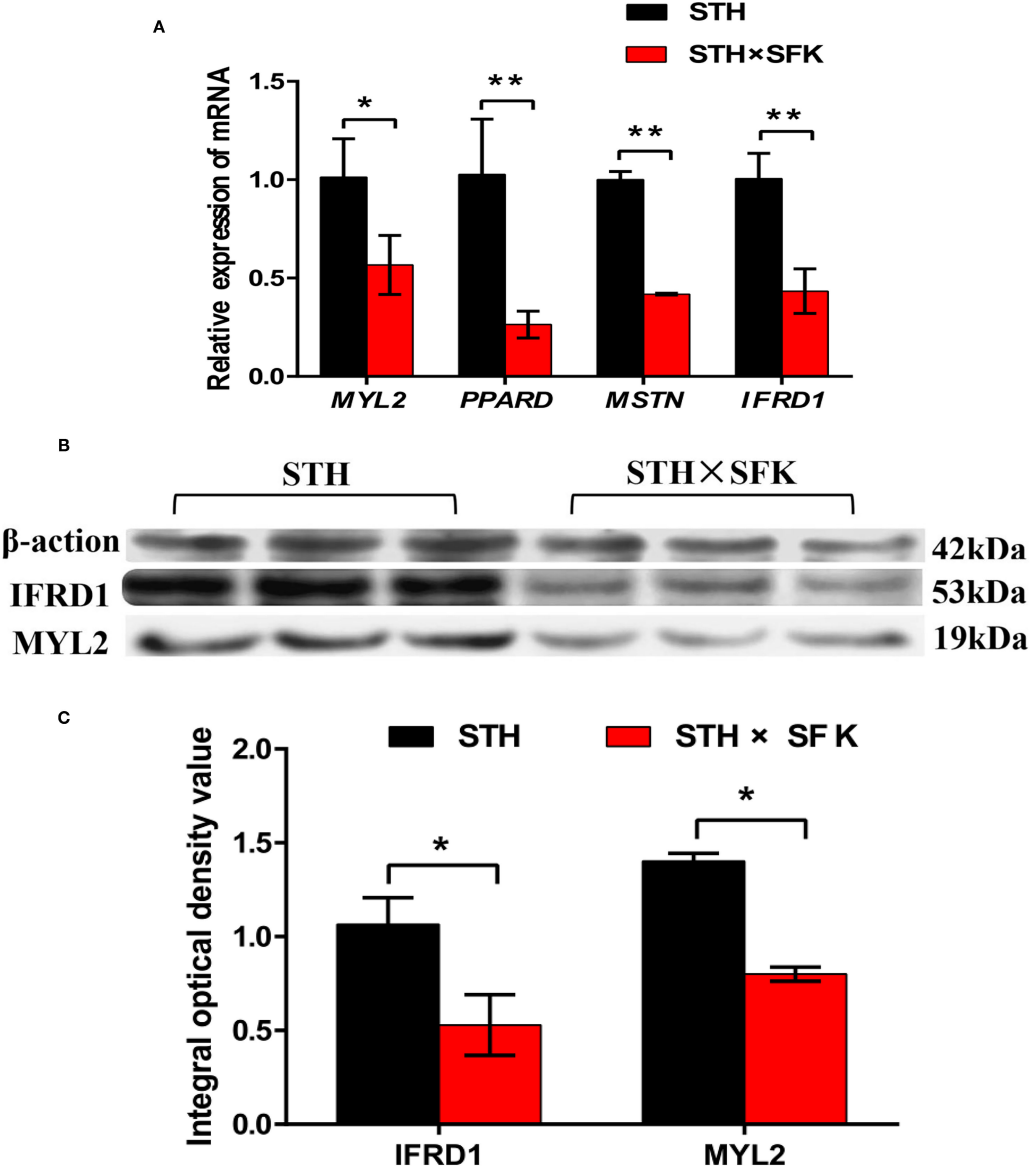
Validation of target genes and proteins of *longissimus dorsi* muscle of STH sheep population and STH×SFK sheep population. **(A)** The mRNA expression levels of 4 DEGs (*MYL2, PPARD, MSTN* and *IFRD1*) were detected by RT-PCR. *Represents *P* < 0.05, **represents *P* < 0.01. **(B)** Two significantly different proteins (IFRD1 and MYL2) were detected by Western blotting. **(C)** Analyze the integrated optical density values of IFRD1 and MYL2 by Graphpad Prism software. *Represents *P* < 0.05.

The *IFRD1, PPARD, MSTN*, and *MYL2* genes selected for sheep growth and meat quality were verified via RT-PCR and WB (Figure 5). The results showed that compared with STH, the expression levels of *IFRD1, PPARD, MSTN*, and *MYL2* genes in the STH×SFK population were significantly down-regulated (*P* < 0.01) (Figure 5A); the expression levels of IFRD1 and MYL2 proteins were also found to be significantly down-regulated (*P* < 0.05) (Figures 5B,C). The trends of RT-PCR and WB results were consistent with those of the transcriptome, indicating that the transcriptome sequencing data were reliable (Supplementary Table 1). It was showed that the expression of such genes could regulate the growth and meat quality of sheep.

### Immunofluorescence Results

**Figure 6:**
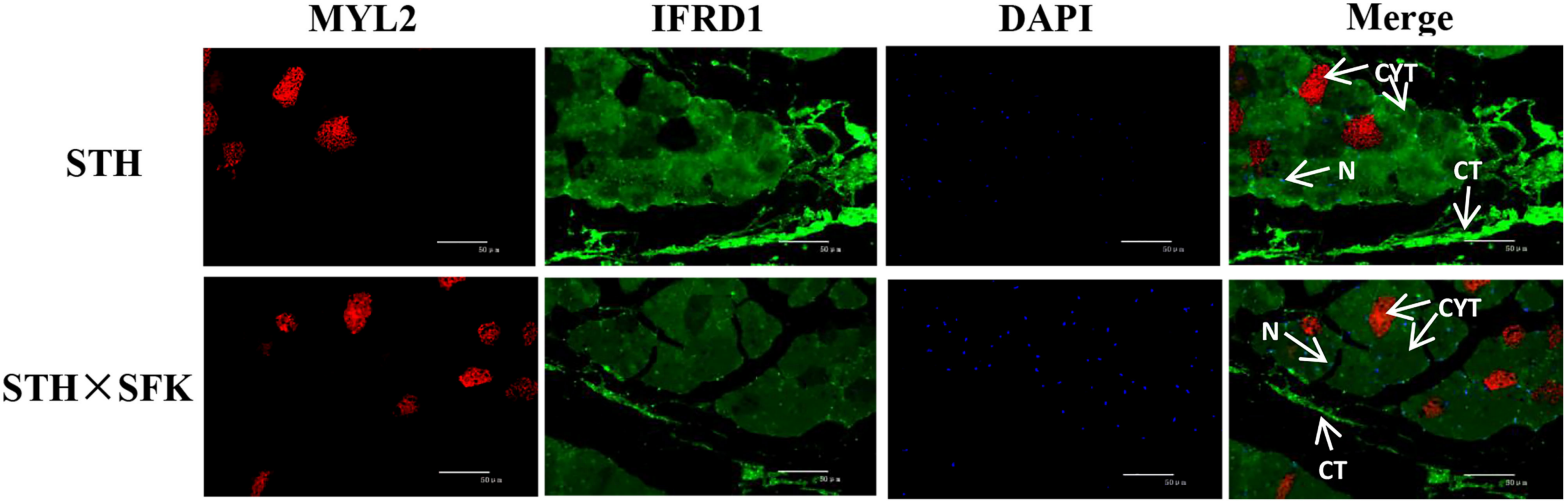
Expression location of MYL2 and IFRD1 protein in STH and STH×SFK *longissimus dorsi* muscle (IF×400). CYT, Cytoplasm; N, Nucleus; CT, Connective tissue.

Immunofluorescence staining of paraffin-embedded sections was performed to understand the localization of IFRD1 and MYL2 proteins in the *longissimus dorsi* tissues of the STH and STH×SFK sheep populations (Figure 6). The results showed that the IFRD1 protein was mainly expressed in the cytoplasm and nucleus, and the MYL2 protein was mainly expressed in the cytoplasm.

## Discussion

Lamb meat is rich in protein (1), and its intake can help supplement calcium and iron levels; additionally, it presents with a low cholesterol content with tender meat quality, rendering increased popularity among consumers. With improvements in living standards, the requirements for the quality of mutton continue to increase. Therefore, it is of considerable significance to study the growth characteristics and meat quality of sheep. The growth and development of sheep and meat quality are not only affected by external factors (such as genetics, environment, and feeding management) but are also affected by the regulation of multiple genes. From a genetic perspective, crossbreeding can help improve sheep growth and meat quality to a certain extent, and this technology has also been widely used in farms; however, a few studies have been conducted at the molecular level. The identification of genes whose expression levels affect sheep growth and meat quality is imperative at the transcriptome level and further research is warranted.

This study compared the production performance and meat quality of crossbred F1 sheep populations. Our results showed that the STH×SFK crossbred F1 was better than the STH sheep, indicating that crossbreeding was beneficial for improving sheep production performance and meat quality. In terms of growth and development, it may be attributed to the inherited characteristics of small-tailed Han sheep, such as a rapid rate of growth and a tall physique, while in terms of slaughter performance, it may be attributed to the inherited characteristics of Suffolk that presents with a better meat production performance (5). The results of H&E staining showed that compared with the STH sheep population, the STH×SFK sheep population was underdeveloped with connective tissue composed of endomysium and perineum. It is generally believed that the perimysium has a greater influence on the tenderness of the meat than the endomysium. The connective tissue contains collagen fibers, and the collagen in the collagen fibers is also an important factor affecting muscle internal strength, and the collagen content is negatively correlated with the tenderness of the meat (24), indicating that the meat quality of the STH×SFK sheep population in this experiment is better. To further reveal this difference, the transcriptome was selected to study the mechanism comprehensively, and transcriptome sequencing analysis of the *longissimus dorsi* muscle of sheep was performed to obtain data on a substantial number of DEGs. The expression of such genes may affect the growth and development of sheep and may trigger changes in meat traits through regulatory effects. Using STH as a control, the STH×SFK crossbred F1 presented with a total of 560 DEGs (377 up-regulated and 183 down-regulated), and bioinformatics analysis revealed that the Ras signaling pathway and the Rap1 signaling pathway were significantly enriched and played roles related to sheep growth and meat quality. The Ras signaling pathway is a MAPK pathway that can be widely activated. Under normal circumstances, Ras binds to GDP in the body. After activating Ras, the MAPK kinase in the cytoplasm is phosphorylated and then activated to enter the nucleus, mediating the expression of specific proteins in the cell, and participates in the regulation of cell proliferation, differentiation, and other functions, and exerts a certain control effect on the types of muscle fibers. Murgia et al. found that after denervation, slow-type MyHC could be induced via continuous activation of Ras and the composition of muscle fibers in the soleus muscle changes (25). Studies conducted by Roth and other researchers demonstrated that the Ras-Raf-MEK1/2-ERK1/2 signaling pathway in the MAPK pathway could upregulate the expression of slow-type fibers MyHC while inhibiting the expression of fast-muscle subtypes (26). Further studies have shown that MAPK phosphatase-1 (MKP1) promotes dephosphorylation and inactivation of MAPK in the nucleus, a mechanism which can effectively promote the conversion of slow-type muscle fibers into fast-type muscle fibers. Rap1 is a GTPase protein and studies have shown that Rap1 protein is mainly produced in the muscle cell domain of mice and undergoes modification to form neuromuscular and tendon connections (27). Additionally, Rap1 signaling can exert a certain effect on the β-adrenergic signaling pathway; studies have also shown that it plays a vital role in the growth and development of skeletal muscle (28). The interaction between the signaling pathways activated by hormones and growth factors is the mechanism by which skeletal muscle undergoes differentiation and maturation. The results indicated that the differences in the growth, development, and meat quality of the *longissimus dorsi* between the two sheep populations may be affected by the DEGs of these pathways.

Transcriptome sequencing aided the identification of four down-regulated genes (*IFRD1, PPARD, MSTN*, and *MYL2*). Among them, FPKM ≥ 0.1 was used as the expression threshold for judging genes, and meaningful genes were screened (22). This finding has also been confirmed in few studies. For example, *MSTN*, also known as growth and differentiation factor 8 (*GDF8*), is a member of the transforming growth factor-beta (*TGF-*β) superfamily. It is a secreted glycoprotein that is expressed in all tissues of the animal body, but its expression is most significant in skeletal muscle (29). Several studies have shown that *MSTN* is not only involved in regulating muscle growth and development but is also related to fat synthesis and decomposition. *MSTN* knockout not only increases the rate of meat production of livestock and poultry but also reduces fat content (30), which exerts a certain impact on meat quality traits. This study found that the *MSTN* gene is involved in growth (GO: 0040007), muscle atrophy (GO:0014889) and muscular system processes (GO: 0003012). Studies have shown that *MSTN* transgenic mice exhibit muscle atrophy, and the number of muscle fibers and the muscle fiber area are significantly reduced (31). Qian et al. (32) found that compared with normal pigs, the area of the eye muscles of the gene-edited pigs was significantly increased, the fat content was significantly reduced, and the thickness of the back and waist was significantly reduced via knockout of the *MSTN* gene in pigs. *PPARD* is a member of the peroxisome proliferator-activated receptor (*PPAR*) family. *PPARs* act as transcription factors that regulate the proliferation of peroxisomes in cells (33–36). *PPARD* can accelerate metabolism and promote the acceleration of fat oxidation by increasing the enzyme activity in the process of fat oxidation metabolism, which is of substantial significance to the breeding of sheep (37). This study found that *PPARD* is mainly involved in biological processes such as growth (GO: 0040007). Ponsuksili et al. (38) found that after knockout of the mouse *PPARD* gene, mice presented with downregulation of fat burning. Meidtner et al. (39) found that the haplotype of *PPARD* was related to the backfat thickness of pigs. The myosin light chain (*MYL*) family includes the basic light chain (*MYL1*) and regulatory light chain (*MYL2*). *MYL2* can regulate the activity of muscle fibers, and the growth and development of skeletal muscles and is related to muscle growth traits (40, 41). This study found that *MYL2* is mainly involved in growth (GO: 0040007), muscle contraction (GO: 0006936) and striated muscle contraction (GO: 0006941). Vicente-Manzanares found that phosphorylation of Ser^19^ and Thr^18^ on the regulatory light chain can increase the activity of ATPase on the myosin heavy chain, can transform the types of muscle fibers, and then affect muscle growth (42). *IFRD1*, also known as *TIS7* (mouse) and *PC4* (rat), is a transcriptional activator or repressor that regulates expression by establishing interaction with HDAC complexes and transcription factors (43, 44). *IFRD1* plays an important role in the regulation of cell proliferation and differentiation by regulating gene expression. Current studies have shown that *IFRD1* is involved in bone formation, skeletal muscle growth, and development (45). This study found that *IFRD1* is mainly involved in biological processes such as growth (GO: 0040007). Vadivelu etal. (45) and other studies have found that knockout of the mouse *IFRD1* gene can delay the regeneration of injured skeletal muscle and change its contraction ability; additionally, it can down-regulate the expression of certain genes related to muscle growth, such as myosin, which in turn exerts a certain effect on muscle growth. Park et al. (46) found that injection of epinephrine in brown and white adipose tissue and the existence of cold conditions can induce the expression of *IFRD1*, which can exert a certain effect on muscles. This experiment also found that the MYL2 protein was expressed in the cytoplasm, and the IFRD1 protein was expressed in the cytoplasm and nucleus. As the cytoplasm is rich in myofibrils, it exerts a certain impact on meat quality. Bouley et al. (47) and other studies found that the MYL2 protein was expressed in muscle fibers in the cytoplasm of goat skeletal muscle. There are also related reports available on the expression and location of IFRD1. For the first time, it was found that the IFRD1 protein was expressed in the nucleus of a cell, and later it was found to be redistributed in the cytoplasm and at sites near the plasma membrane (48, 49), an observation that was consistent with that documented in this experiment. The above-mentioned results indicate that the four genes, namely IFRD1, PPARD, MSTN, and MYL2, may exert a regulatory effect on the growth and development of sheep muscles, which can be combined with sheep breeding to perform selection of good breeds and to provide a theoretical basis for studying sheep genetic breeding.

## Conclusion

In this study, the growth performance, slaughter performance, and meat quality of STH and STH×SFK were compared, and it was found that STH×SFK bust circumference, pre-slaughter live weight, slaughter performance, and marbling score were significantly higher than STH, indicating that the crossbred population was better than the pure breed population. Transcriptome sequencing of STH and STH×SFK *longissimus dorsi* muscle samples was performed, and 560 DEGs were screened, of which 377 exhibited up-regulated and 183 exhibited down-regulated expression levels. Through the GO and KEGG enrichment analysis, four co-expressed genes (*IFRD1, PPARD, MYL2*, and *MSTN*) could be related to sheep growth, development and meat quality. Immunofluorescence results revealed that the IFRD1 protein was mainly expressed in the cytoplasm and nucleus, and the MYL2 protein was mainly expressed in the cytoplasm. In summary, these genes could be used as candidate genes for sheep growth, development and meat quality genetic breeding improvement, laying a foundation for in-depth study of the regulation mechanism of sheep muscle growth.

## Data Availability Statement

The data supporting the results of this study have been provided as Supplementary Material, and other detailed information can be obtained from the corresponding author.

## Ethics Statement

The study was conducted according to the guidelines of the Animal Welfare Committee of the College of Animal Science and Technology of Gansu Agricultural University and approved by the Institutional Review Board (GSAU-AEW-2017-0308).

## Author Contributions

YS, QZ, and SC have made substantial contributions to design conception, material collection, experimental research, manuscript writing, and final version revision. JS and LF participate in experiments and data analysis. All authors have read and agreed to the published version of the manuscript.

## Funding

This research was supported by the Gansu Agricultural University, Gansu Province, China (No. GAU-XKJS-2018-024).

## Conflict of Interest

The authors declare that the research was conducted in the absence of any commercial or financial relationships that could be construed as a potential conflict of interest.

## Publisher's Note

All claims expressed in this article are solely those of the authors and do not necessarily represent those of their affiliated organizations, or those of the publisher, the editors and the reviewers. Any product that may be evaluated in this article, or claim that may be made by its manufacturer, is not guaranteed or endorsed by the publisher.
