# Supplementary material for: Screening of Genes Related to Growth, Development and Meat Quality of Sahan Crossbred F1 Sheep Based on RNA-Seq Technology
Source: Front Vet Sci. 2022 Apr 7;9:831519. doi: 10.3389/fvets.2022.831519 (PMC9021821; doi:10.3389/fvets.2022.831519)
Supplement: Supplementary file 1 [file Data_Sheet_1.docx]

**Table S1 Real-time fluorescence quantitative PCR primer sequence**

| Gene | Primer sequence (5'～3') | Product length /bp |
| --- | --- | --- |
| *MSTN* | F: GGAGAGATTTTGGGCTTGA  R: ACCTTTGGGGTTTGCTTGG | 209 |
| *IFRD1* | F: GGCATGTACCAGGAAGCAGT  R: CTCAGTTGGTGCCTGGGTAT | 250 |
| *MYL2* | F: CTGCTCTTGGGCGTGTG  R: TCTCCGTCTGCGTGGTC | 225 |
| *PPARD* | F: ATAAATACCAGCCCCGGTTC  R: ACTGCAGGGTGAGTTGCTTT | 225 |
| *ACTB* | F: GGCATCCTGACCCTCAAGTA  R: GGGGTGTTGAAGGTCTCAAA | 203 |
| *GAPDH* | F: TGTTTGTGATGGGCGT  R: TCTGGGTGGCAGTGAT | 168 |

**Table S2 Production performance and meat quality differences between STH and STH×SFK sheep populations**

| Indexes | STH | STH×SFK | *P* value |
| --- | --- | --- | --- |
| Height/㎝ | 63.83±1.57 | 63.42±1.50 | 0.133 |
| Body length/㎝ | 70.00±2.45 | 67.42±1.66 | 0.197 |
| Bust/㎝ | 68.67±1.94^B^ | 82.25±2.96^A^ | 0.001 |
| Live weight before slaughter/㎏ | 28.01±2.47^B^ | 40.15±2.58^A^ | 0.001 |
| Carcass weight/kg | 12.73±0.99^B^ | 20.46±1.58^A^ | 0.001 |
| Net-meat weight/㎏ | 9.30±0.79^B^ | 15.14±0.89^A^ | 0.001 |
| Carcass production rate/% | 73.01±0.97 | 74.11±1.94 | 0.406 |
| Net-meat percentage/% | 33.22±0.52^B^ | 37.73±1.03^A^ | 0.001 |
| Meat bone ratio | 3.14±0.08^B^ | 3.36±0.10^A^ | 0.001 |
| Dressing percentage/% | 45.51±1.05^B^ | 50.92±0.96^A^ | 0.001 |
| Loin eye area/cm^2^ | 11.76±0.60^B^ | 14.35±0.96^A^ | 0.001 |
| Back meat thick/cm | 1.64±0.06^B^ | 1.81±0.10^A^ | 0.001 |
| pH值 | 5.56±0.06 | 5.54±0.08 | 0.16 |
| Marbling grade | 1.42±0.38^B^ | 2.50±0.55^A^ | 0.001 |
| Water loss rate/% | 9.77±1.34 | 8.40±2.54 | 0.544 |
| Moist cooking loss/% | 45.79±0.28 | 44.66±2.50 | 0.423 |
| Brightness value/L | 35.93±4.43 | 33.97±2.96 | 0.195 |
| Red value/a | 10.42±0.94 | 10.08±1.83 | 0.930 |
| Yellowness value/b | 9.18±1.06 | 7.68±1.50 | 0.119 |
| Cooking loss/% | 36.70±3.65 | 34.97±5.37 | 0.118 |
| Shear force/N  Note: In peer data, different uppercase letters indicate extremely significant differences (*P*<0.01), and different lowercase letters indicate significant differences (*P*<0.05). | 26.98±2.22^a^ | 24.43±1.21^b^ | 0.012 |

**Table S3 Comparison statistics of sequencing data results in STH and SFK × STH sheep populations**

| Sample | Clean Data/bp | HQ Clean Data/bp | Q30(%) | Unmapped Pair Reads/bp | Unique Mapped Pair Reads/bp | Mapping Ratio/% |
| --- | --- | --- | --- | --- | --- | --- |
| STH1 | 10793742362 | 10738865766 | 91.18 | 17599433 | 50717048 | 74.42 |
| STH2 | 10218998155 | 10167762410 | 91.93 | 13673048 | 52908475 | 79.62 |
| STH3 | 9609088359 | 9558576789 | 91.26 | 12865585 | 50100849 | 79.73 |
| STH4 | 9864384240 | 9805315272 | 91.02 | 13328747 | 51297178 | 79.54 |
| STH5 | 10114592547 | 10058306387 | 91.02 | 14635166 | 51240912 | 77.96 |
| STH6 | 11069022361 | 11004278518 | 90.43 | 15991408 | 55907174 | 77.92 |
| STH-SFK1 | 9344370598 | 9297661542 | 90.68 | 13295504 | 47779108 | 78.37 |
| STH-SFK2 | 9009665041 | 8944580280 | 91.13 | 11698538 | 47185388 | 80.28 |
| STH-SFK3 | 10583160640 | 10507802858 | 91.07 | 16075381 | 52258873 | 76.66 |
| STH-SFK4 | 10298328487 | 10230918080 | 90.99 | 14837511 | 52070502 | 78.04 |
| STH-SFK5 | 9838651377 | 9757854467 | 90.89 | 15813121 | 47518955 | 75.19 |
| STH-SFK6 | 9814835229 | 9747763445 | 90.95 | 14344011 | 49362565 | 77.71 |


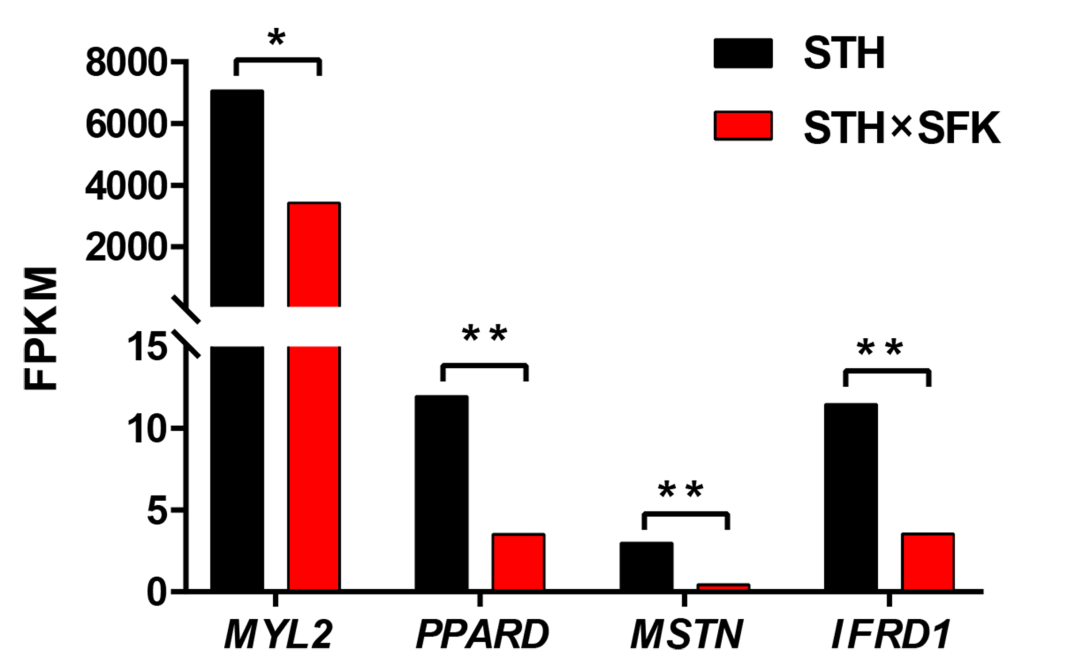


Fig. S1. Transcriptome analysis detected the mRNA expression levels of 4 genes.

*Represents P<0.05, **represents P<0.01
